# The need for adaptable global guidance in health systems strengthening for musculoskeletal health: a qualitative study of international key informants

**DOI:** 10.1186/s41256-021-00201-7

**Published:** 2021-07-14

**Authors:** Andrew M. Briggs, Joanne E. Jordan, Deborah Kopansky-Giles, Saurab Sharma, Lyn March, Carmen Huckel Schneider, Swatee Mishrra, James J. Young, Helen Slater

**Affiliations:** 1 Curtin School of Allied Health, Curtin University, Perth, Australia; 2 HealthSense (Aust) Pty Ltd, Melbourne, Australia; 3 Department of Research, Canadian Memorial Chiropractic College, Toronto, Canada; 4 Department of Family & Community Medicine, University of Toronto, Toronto, Canada; 5 Department of Physiotherapy, Kathmandu University School of Medical Sciences, Dhulikhel, Nepal; 6 Department of Rheumatology, Royal North Shore Hospital, Sydney, Australia; 7 Sydney Musculoskeletal, Bone & Joint Health Alliance, Faculty of Medicine and Health, University of Sydney, Sydney, Australia; 8 Menzies Centre for Health Policy and Economics, Faculty of Medicine and Health, University of Sydney, Sydney, Australia; 9 Center for Muscle and Joint Health, Department of Sports Science and Clinical Biomechanics, University of Southern Denmark, Odense, Denmark

**Keywords:** Global health, Disability, Rehabilitation, Musculoskeletal, Strategy, Systems strengthening

## Abstract

**Background:**

Musculoskeletal (MSK) conditions, MSK pain and MSK injury/trauma are the largest contributors to the global burden of disability, yet global guidance to arrest the rising disability burden is lacking. We aimed to explore contemporary context, challenges and opportunities at a global level and relevant to health systems strengthening for MSK health, as identified by international key informants (KIs) to inform a global MSK health strategic response.

**Methods:**

An in-depth qualitative study was undertaken with international KIs, purposively sampled across high-income and low and middle-income countries (LMICs). KIs identified as representatives of peak global and international organisations (clinical/professional, advocacy, national government and the World Health Organization), thought leaders, and people with lived experience in advocacy roles. Verbatim transcripts of individual semi-structured interviews were analysed inductively using a grounded theory method. Data were organised into categories describing 1) contemporary context; 2) goals; 3) guiding principles; 4) accelerators for action; and 5) strategic priority areas (pillars), to build a data-driven logic model. Here, we report on categories 1–4 of the logic model.

**Results:**

Thirty-one KIs from 20 countries (40% LMICs) affiliated with 25 organisations participated. Six themes described contemporary context (category 1): 1) MSK health is afforded relatively lower priority status compared with other health conditions and is poorly legitimised; 2) improving MSK health is more than just healthcare; 3) global guidance for country-level system strengthening is needed; 4) impact of COVID-19 on MSK health; 5) multiple inequities associated with MSK health; and 6) complexity in health service delivery for MSK health. Five guiding principles (category 3) focussed on adaptability; inclusiveness through co-design; prevention and reducing disability; a lifecourse approach; and equity and value-based care. Goals (category 2) and seven accelerators for action (category 4) were also derived.

**Conclusion:**

KIs strongly supported the creation of an adaptable global strategy to catalyse and steward country-level health systems strengthening responses for MSK health. The data-driven logic model provides a blueprint for global agencies and countries to initiate appropriate whole-of-health system reforms to improve population-level prevention and management of MSK health. Contextual considerations about MSK health and accelerators for action should be considered in reform activities.

**Supplementary Information:**

The online version contains supplementary material available at 10.1186/s41256-021-00201-7.

## Background

Global leadership to support country-level health systems strengthening to arrest the burden of non-communicable diseases (NCDs) is imperative [1–4]. Non-communicable diseases are well recognised as the major contributors to the global burden of disease, as measured by disability-adjusted life years (DALYs). In 2019, NCDs reflected 64% of the total global burden [5], while in low and middle-income countries (LMICs), NCDs and injuries accounted for 66% of the DALYs – a dramatic shift from 39% in 1990 [5]. While the urgency for health systems to respond to the health, social and economic burden associated with NCDs is reflected in targets for the Sustainable Development Goals (SDGs) and in major international reviews and initiatives to steward system reform efforts [1, 6–8], Universal Health Coverage for NCDs lags well behind that for communicable, maternal, neonatal, and nutritional (CMNN) diseases, particularly in middle-income economies [9].

The health and socioeconomic burden of musculoskeletal (MSK) health impairment mirrors, and often eclipses, that of other NCDs. As a group of NCDs, established MSK health conditions are the leading cause of disability worldwide, representing 17% of the global years lived with disability (YLDs) in 2019, with the rate of MSK-attributed YLDs per 100,000 population increasing by 23% since 1990 [5]. MSK conditions are the highest contributors to the global need for rehabilitation services across lifecourse [10] and a major contributor to the burden of chronic pain [11]. Low back pain remains the single leading cause of disability worldwide since 1990 and the leading cause in most countries, irrespective of economic development [5]. Neck pain, osteoarthritis and other MSK disorders feature among the top 20 conditions contributing to the global burden of disability. The burden of disease related to MSK health impairment increases substantially when considering disability related to MSK health impairments outside the context of NCDs, including MSK injury and trauma, such as road traffic accidents, falls and industrial accidents, and where persistent pain manifests through MSK conditions [5, 12, 13]. Critically, MSK health conditions are relevant across the life-course [14]. They frequently feature in co- and multi-morbidity health states for NCDs [15, 16], are a risk factor for other NCDs [17, 18] and are a key determinant of intrinsic capacity associated with healthy ageing [19]. On a background of rapid global ageing and an increasing prevalence of NCDs and associated risk factors, the burden of disability related to MSK health conditions will continue to rise and increasing poverty and disability in adults [18, 20] and children [21] and rising societal costs will likely ensue [22]. The COVID-19 pandemic has brought this health and socioeconomic challenge into even sharper focus. NCDs and their modifiable risk factors increase the risk of COVID-19-related illness and mortality [23–25], and morbidity for older people inclusive of MSK function [26]. Concurrently, social threats associated with COVID-19 are proposed to exacerbate the experience of persistent pain [27]. This has implications for LMICs, as MSK conditions are among the leading causes that account for more than 75% of disease burden for NCDs and injuries for the poorest billion people aged 5–40 years and greater than 40 years of age [1].

This significant and escalating burden raises the question: *why is MSK health not prioritised within global or national health system strengthening efforts commensurate with its burden of disease* [3, 28–32]*?* This lack of prioritisation highlights a lost opportunity to positively impact the global burden of disability [33]. ‘Calls to Action’ for global responses to the health and economic burden attributed to MSK conditions have been made for some time, preceding, during and following the Bone and Joint Decade 2000–2010 [34], with the need for global action on MSK health a key finding from GBD 2019 [4]. While much progress was achieved through the Decade through raising awareness about MSK disease burden, sustained and systematic health system strengthening responses are lacking and disability continues to rise [33]. Most calls have focused on ‘*what’* needs to be done [18, 31, 32, 35–38]. Few have addressed ‘*how’* this could be achieved within dynamic health ecosystems, although recent frameworks have been proposed for creating value-based health systems [39], and determinants of political priority for global health initiatives [40, 41].

The Global Alliance for Musculoskeletal Health (G-MUSC) called for a strategic global response to address health systems strengthening for MSK health. In response to that call, this research aimed to explore how a global response might be framed through in-depth consultation with the global MSK community and other multi-sectoral stakeholders (including patients and advocacy organisations). Specifically, we aimed to explore contemporary challenges and opportunities at a global level relevant to systems strengthening for MSK health, as identified by key informants (KIs), including patients. This study forms part of a broader program of research that will inform a blueprint for a global response to improving the prevention and management of MSK health.

## Methods

### Design

A qualitative study using individual, semi-structured interviews with KIs was undertaken in 2020. Approval to undertake the study was granted by the Human Research Ethics Committee of Curtin University, Australia, and in accordance with the Declaration of Helsinki. All participants provided informed consent. An External Steering Group, appointed by the G-MUSC executive was established to oversee the stages of the project and offer strategic advice. The Steering Group had no role in data collection, analysis, interpretation, reporting or decisions on publication. The manuscript is reported in alignment with the COREQ-32 and GRIPP2-sf checklists (Supplementary files 1 and 2 [42, 43],).

### Sampling

To achieve diversity in the important domains of actors, ideas, contexts and characteristics, identified by Shiffman and Smith (Table 1 [40]), KIs were purposively sampled across six categories deemed relevant to the MSK global community.

**Table 1:** Purposive sampling categories

• A President/Chair, Vice President or appropriately delegated senior-level official (e.g. leader of a special interest group or subcommittee) of an international or global clinical/professional organisation relevant to MSK health and/or persistent pain care and having held this post for at least 12 months. • A President/Chair, Vice President or appropriately delegated senior-level official of an international or global advocacy (including patient advocacy) organisation relevant to MSK health, persistent pain care, injury, ageing, NCDs, or health systems strengthening and having held this post for at least 12 months. • An official of the World Health Organization (WHO) with a scope of work relevant to MSK health, ageing and lifecourse or NCDs and having held this post for at least 12 months. • A senior officer in a national Ministry of Health having held a position for at least 12 months that includes international activities in health systems strengthening efforts (i.e. beyond a single national context). • A thought leader defined by the publication of at least 3 peer-reviewed journal papers or health policies in the last 5 years that have a focus on health system reform or health policy relevant to MSK health or persistent pain care. • A person with a lived experience of an MSK health condition and/or persistent MSK pain for more than 5 years.	

To ensure diversity across clinical disciplines, sectors, geographies and economic development, a maximum heterogeneity sampling approach to identify KIs was adopted. In addition to satisfying one of the six eligibility criteria (Table 1), KIs needed to be at least 18 years of age and able to speak and read English. Other than the category of ‘thought leader’, KIs were intentionally sampled as affiliates or representatives of organisations to enable results to be reflective of broader perspectives, beyond just those of the individual, consistent with earlier aligned research [44]. However, the data presented, do not necessarily reflect the endorsed views of the organisations represented.

KIs were invited to participate via personal email sent from the G-MUSC office (Sydney, Australia) on behalf of the research team, between the period 5th June and 22nd July 2020. The invitation outlined the purpose of the study and included a link to an online eligibility screening survey, consent form and a demographics questionnaire powered by Qualtrics™ (Provo, UT, USA). KIs were sampled across four sequential rounds to ensure balanced representation across sampling categories and diversity criteria, as well as the outcomes of interim analyses.

### Interview schedule development

A semi-structured interview schedule was iteratively developed by the multi-disciplinary research team to explore KIs’ perceptions relating to:

1. The current state of MSK health globally (both prevention and management).
2. Actions needed at a global level to address MSK healthcare and strengthen health systems.
3. The potential value of a global strategy to improve prevention and management of MSK health.
4. Requisite components for a global strategy, including goals.
5. Priorities and opportunities for improving prevention and management of MSK health aligned with the six objectives from the *WHO Global Action Plan for Prevention and Control of NCDs (2013–2020* [6]).

MSK health was defined as any condition affecting the MSK system, MSK pain and MSK injury or trauma.

### Data collection

#### Pilot phase

To test that the interview questions were clear and comprehensible and eliciting relevant responses, the interview schedule was piloted with three KIs (one patient, two health professionals) from different countries where English was not the native language. After analysing these three pilot transcripts, the schedule was revised and finalised (Supplementary file 3).

#### Interviews

Data collection was undertaken between June and August 2020. A highly-experienced qualitative research fellow (JEJ) conducted two-thirds of the interviews following a standard approach determined *a priori*. The remainder were conducted by AMB and HS, both MSK health researchers experienced in qualitative methods. One week before the interview, KIs received the interview questions to ensure sufficient time to consider their responses and allow consultation with their organisation(s). Interviews were conducted privately in English by telephone or via a secure videoconferencing platform and audio-recorded for transcription, supported by field notes. A verbatim transcript was sent to each participant to ensure that it was an accurate record of the discussion, and to provide an opportunity to add further comments/information. An initial 18 interviews were conducted and formatively analysed in June-early July. In order to explore emerging concepts further, another 9 interviews were undertaken in mid to late-July and a further 4 interviews in late July/early August, with recruitment ceasing at that point (*n* = 31), as no new concepts had emerged.

#### Data processing and analysis

Interview transcripts were analysed in three sequential phases, aligned to sampling rounds, using a grounded theory method [45, 46].

In phase 1, to generate codes from the data (JEJ), each of the 18 transcripts was analysed line by line (open coding). This analysis was performed concurrently with data collection. After the first 5 transcripts had been analysed, a list of initial codes linked to corresponding data was inductively derived (JEJ) and verified by a second analyst (AMB). These initial codes were then applied and developed further with the next 5 transcripts. After analysing 10 transcripts, logical groupings of codes (axial coding) were developed by JEJ and AMB. Further inductive analysis was undertaken for the next 8 transcripts with constant comparative analysis between codes and the raw data and memos guiding the development of a framework of categories (selective coding) that identified:

1. Context: a contemporary contextual factor associated with MSK health at the global level.
2. Goal(s): suggested ambitions or targets for a global strategy on MSK health.
3. Guiding principles: concepts or approaches that should underpin all activities or actions within a strategy.
4. Accelerators: processes or supports that enable action on strategic priority areas.
5. Strategic priority areas or ‘pillars’: components or groups of actions important for a contemporary global strategy on MSK health.

Following selective coding, to verify the categories and codes developed, three members of the research team (DKG, SS, HS) then independently analysed four transcripts. The framework of codes and categories were further refined through discussion. Throughout this process, further memos were generated to assist initial thinking and conceptualising of a logic model.

In phase 2, the next 9 transcripts were analysed using the refined codes established in phase 1 of the analysis, as well as generating new codes where new information was identified. The categories developed in phase 1 were then reviewed in light of the codes and data added from phase 2. Categories were then further refined, merged or changed to reflect different dimensions that had emerged from the phase 2 data. This refinement was undertaken via a series of meetings between two analysts (JEJ, AMB). Memos were generated to iterate and reconceptualize the logic model.

In phase 3, the last 4 transcripts were analysed using the refined codes and only two new codes were generated. The categories refined in phase 2 were then reviewed and minor revisions to the descriptions of categories made. No new categories were developed.

Once categories and codes were finalised, the logic model for a global MSK strategy was finalised to ensure that all the categories were conceptually and meaningfully linked and to provide further classifications within categories (i.e. sub-categories), where appropriate. The logic model was reviewed and refined by the three members of the research team who had previously verified phase 1 analysis outcomes.

## Results

### Sample characteristics

**Fig. 1:**
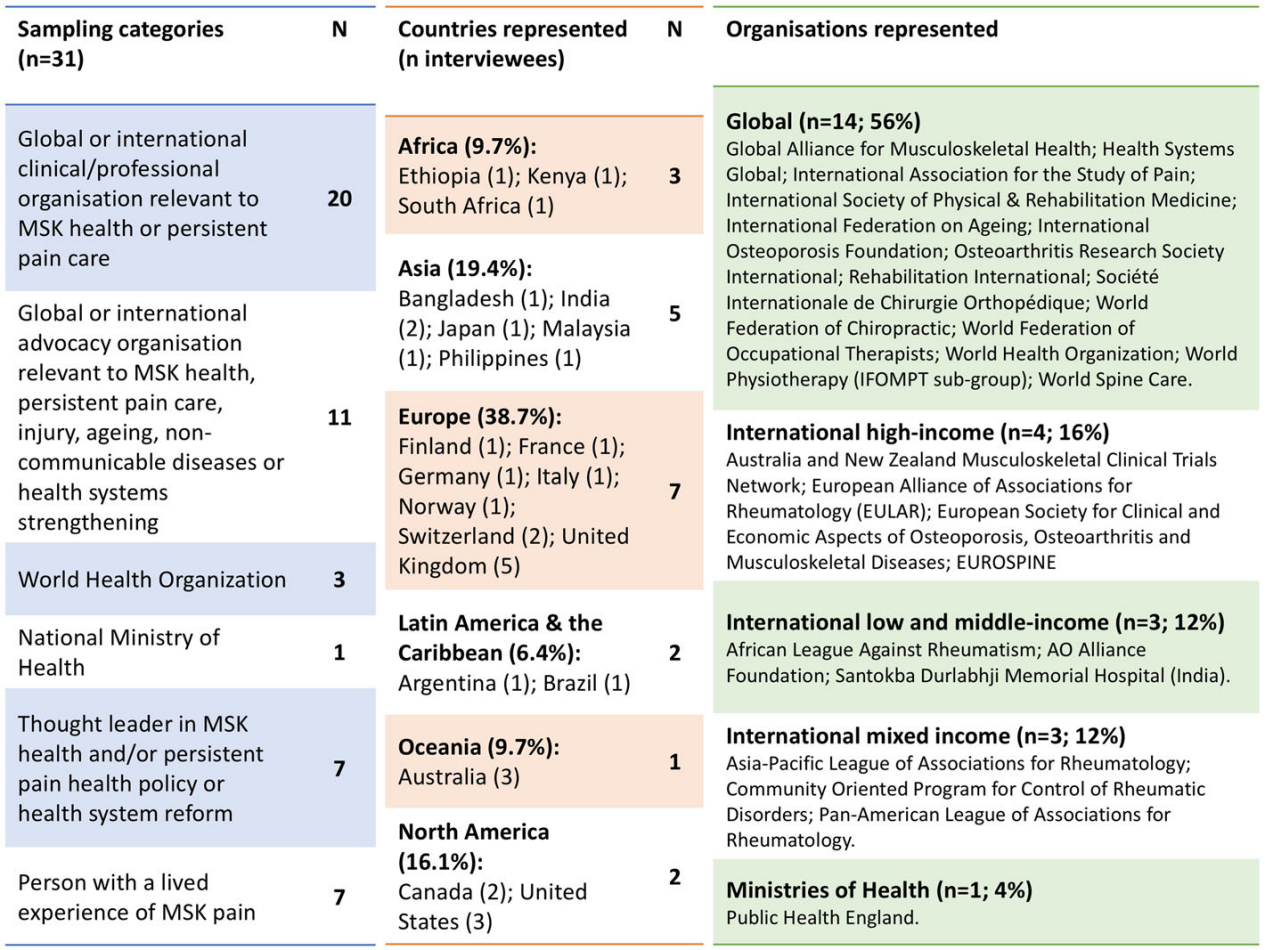
Distribution of the sample across sampling categories (left panel) and geographies (middle panel). Organisations represented are listed in the right panel. While KIs identified as representing these organisations, the views expressed are not necessarily official statements from the organisations

31 KIs (45%, female) from 20 countries (40% LMICs based on the World Bank list of economies, June 2020) with a mean (SD; range) age of 57.9 (10.8; 41–77) years and 30.4 (11.2; 6–53) years of experience in healthcare participated and 2 declined. Collectively, the informants represented 25 organisations (Fig. 1). Across the informants, 4 (13%) were patient representatives of international or global organisations, while 7 (23%) had a lived experience of a MSK health condition/persistent MSK pain for a mean (SD; range) duration of 27.3 (17; 10–52) years. Of the 31 participants, 22 (71%) were registered clinicians, including: rheumatologists (*n* = 5), orthopaedic surgeons (*n* = 3), physiotherapists (*n* = 3), chiropractors (*n* = 2), physical medicine and rehabilitation physicians (*n* = 2), public health physicians (*n* = 2), family medicine physician (*n* = 1), emergency medicine physician (*n* = 1), occupational therapist (*n* = 1), neurologist (*n* = 1) and paediatric rheumatologist (*n* = 1). Mean (SD) interview duration was 36 (9) minutes (range: 23–55 min).

### Logic model

**Fig. 2:**
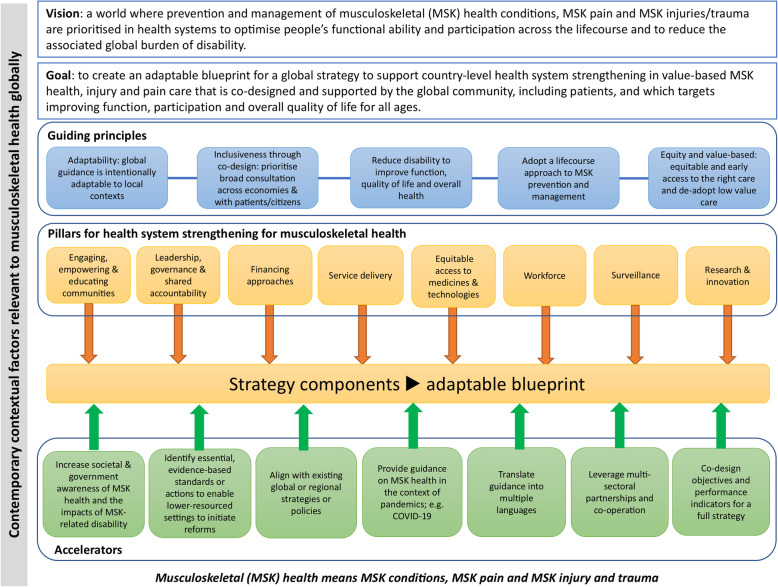
Empirically derived logic model for a global strategy for musculoskeletal health. The focus of this paper is on contemporary and contextual factors relevant to MSK health globally, guiding principles and accelerators. The pillars for health systems strengthening for MSK health are described in an aligned manuscript [47]. Terminologies are aligned with those described by Menear et al. [39] for leaning health systems

The qualitative data were used to create a logic model, consisting of five components, aligned to data coding and categorisation (Fig. 2). The Vision of the logic model was adapted from the existing G-MUSC vision. In order to report findings in a comprehensive and detailed manner and to establish clear context for the components of a strategy, this paper focuses on: contemporary contextual factors (category 1); goals (category 2); guiding principles (category 3); and accelerators (category 4) - each described sequentially in the results. The specific pillars to be considered within a global MSK strategy (category 5) are reported comprehensively in an aligned manuscript [47]. In brief, however, eight pillars were identified (category 5), with each incorporating a number of components that could meaningfully inform a global strategy blueprint for MSK health (Fig. 2).

### Category 1: Contemporary contextual considerations relevant to musculoskeletal (MSK) health globally

There was strong support across KIs for a global strategy to catalyse and steward country-level health system strengthening responses for MSK health, inclusive of MSK conditions, MSK pain and MSK injury and trauma. Six key themes were identified, reflecting important contextual factors regarding challenges and opportunities in the current global MSK health landscape that would need to be considered in the formulation of any strategy for improving the prevention and management of MSK health. These included:

1. MSK health is afforded a relatively lower priority status compared with other health conditions and is poorly legitimised.
2. Improving MSK health is more than just healthcare.
3. Global guidance is needed for country-level health system strengthening.
4. COVID-19 will have an impact on MSK health globally and opportunities for health systems strengthening.
5. There are multiple inequities associated with impaired MSK health.
6. Service delivery for MSK health is characterised by multiple complexities.

Table 2 contains a summary of all six contextual themes identified by the KIs. The most prominent themes to emerge are discussed in further detail below.

**Table 2:** Contextual considerations relevant to musculoskeletal (MSK) health globally, relevant to Category 1 of the logic model

Theme	Sub-themes	Illustrative quote(s)
**1. MSK health is afforded a relatively lower priority status compared with other health conditions and is poorly legitimised**	1.1 MSK conditions are, and historically have been, considered a relatively lower health priority across all levels of society compared with other health conditions more closely associated with mortality and urgency.	*“Certainly, in my region in Africa, I think the communicable diseases seem to take a priority and in terms of the NCDs, the cardiovascular diseases and perhaps the cancers seem to be on top of the list of priorities.”* (ID11) *“I think that musculoskeletal conditions are not directly life-threatening diseases, so the importance of musculoskeletal conditions has been underestimated. I think that lower back pain and knee osteoarthritis are two major targets in musculoskeletal conditions, but many people feel that conditions such as knee osteoarthritis would be far less important compared to cancer or cardiovascular disease.”* (ID1) *“I think that musculoskeletal disorders still remain rooted at the foot of the priority ladder when it comes to NCDs.”* (ID7) *“My general thinking about musculoskeletal conditions is it seems like it is one of the forgotten or sidelined issues. The prevalence is really, really high and the contribution to disability is really high, but the focus given to the prevention and management of musculoskeletal conditions doesn’t really match …*” (ID30)
	1.2 MSK pain and specific MSK conditions are often poorly understood, recognised, measured, treated and legitimised in policy, practice and in community attitudes.	*“The other issue that we’ve got particularly around inflammatory arthritis, you can’t see it anymore. Because we’ve come such a long way with the medications and the treatment options available, you don’t see the disfigurement, you don’t see the old people hunched over as much as you used to. You don’t see it, it’s become an invisible disease that is still as debilitating, it is still as painful, but because you don’t see it, it’s not front of mind, it’s not challenging people’s understanding of how horrible it is anymore, which has actually borne out a whole new issue for people who are dealing with this. Because it’s become an invisible disease, people don’t accept that there’s anything wrong with you.”* (ID12) *“So, in terms of* [MSK pain] *management, we haven’t done that at all for the average adults. There is pain management for kids in the WHO but there is no pain management for adults, including low back pain which is a leading cause of disability. It’s very important to address not only the pharmacological interventions.”* (ID24)
	1.3 MSK health and pain care are relatively lower priorities for investment in service delivery, workforce and research by nations and donors.	*“… countries with limited health budgets will still prioritise life-saving health measures and, as I said before, musculoskeletal disorders don’t kill enough people to make the government sit up and listen. I think that is one of the bottom lines. So while we know that they cause widespread disability, huge disability, they don’t cause death, and I think some of the funding for research and innovation is likely to be compromised for that reason.”* (ID7) *“The other immediate thought that I have, and that is probably across the board, is the resourcing. I feel that the whole musculoskeletal pathway right through from talking about prevention to rehabilitation compared to some of the other long-term conditions is probably very poorly resourced, especially at the front end on the prevention side, that is.”* (ID17) *I can boldly say that it’s not a priority health issue like, for example, in Ethiopia and other African countries. It’s not a priority health issue...other health issues like, for example, communicable diseases like HIV/AIDS and tuberculosis. The government budget, more budget goes to communicable diseases and these non-communicable diseases are not really considered a top priority issue. Currently, there are some changes of course, but still there is a huge gap. Like if you see the health policy and the health strategy, they give more weight to the communicable diseases than to the non-communicable diseases.”* (ID30)
**2. Improving MSK health is more than just healthcare**	2.1 There is inadequate inter-ministerial cooperation and inadequate integration of MSK health into public policy to drive improvements in prevention and management of MSK health impairments. MSK health is relevant beyond healthcare and extends to other important areas such as industry, transport and infrastructure.	*“So I would say at the national leadership level that a move like that is important. We have to get rid of the elephant in the room, it has to be treated at the same level because we are number one on the years lived with disability scores from the global burden of disease. So, it has to be included in every national plan about public health and any action to reduce absence from work* etc. *So that’s very important.”* (ID2)
	2.2 Built environments are often not conducive to preventing MSK health impairment, managing MSK health conditions and enabling participation and health promotion by people with MSK conditions.	*“You may not really want to ever get out of your home because it’s really challenging to move around. Then you find where the road systems have improved, like in the capital city here we now have nice, really beautiful highways, but the highways actually have limited places where you can cross on foot. So, if you’re physically challenged and the vehicles are cruising at a high speed - or if there’s a crossing, it is very, very far away, it is so far that you can’t walk that distance. These are not major, but they are major to the quality of life.”* (ID3)
**3. Global guidance is needed for country-level health system strengthening**	3.1 There is a need for a global strategy to improve prevention and management of MSK health.	*“Because NCDs are not uniform, they don’t have uniform needs, so a specific MSK policy which will cater now for all the needs, like the issue of mobility, the issue of access, yes and that I think can be really actually promoted at a global level and then it’s for the individual governments worldwide to decide what level they’re able to adopt, according to their resources and political will.”* (ID3)
	3.2 External framing of MSK health with a focus on cost to governments and return on investment is needed.	*“I think if you look at the numbers, the costs, they are huge, they are massive, and it’s comparable with major investments for defence. You can reframe the costs and illustrate them by this costs the same every year as it costs our marines to buy these four ships or things like that.”* (ID2) *“I think also it’s fair to say that government action tends to follow economic pressure and if governments really feel the pressure of economic harm arising from a particular activity within society or a burden on society like musculoskeletal disorders then that may prompt them to take better action, but because they’re not asking the questions, possibly because they know what the answers are going to be, it gives them an excuse not to invest. I wonder whether that’s an issue as well.”* (ID7)
	3.3 Global leadership from the WHO in prioritisation of MSK conditions, through the development of a strategy, action plan or guideline, is essential to catalyse a global response to the burden of disease, particularly in LMICs and the activities of global clinical organisations.	*“Yes, I believe that if that top-level strategy came from the WHO and trickled down - actually, maybe “trickled” is not the right world to use here. I think it needs to come down more forcefully, more authoritatively. It needs to come down through all the right channels with a proper and co-ordinated flow.”* (ID31) *“I think there definitely needs to be a concerted effort at governmental level to prioritise MSK disorders within health systems globally. … without a global strategy the management of MSK disorders will continue to be suboptimal.”* (ID7)
**4. COVID-19 will have an impact on MSK health globally and opportunities for health systems strengthening**	4.1 The global COVID-19 pandemic will likely increase financial and racial inequities in healthcare and reduce prioritisation of MSK health and pain care by governments on a background of a likely increase in MSK burden of disability.	*“Like, for example, if you look at home rehabilitation services for people with disabilities, now because of COVID we are not able to provide home-based rehabilitation services because most rehabilitation services require physical contact. So, because of this, most rehab in institutions in Addis Ababa and some African countries, they’re almost not functioning at this point. This situation worsens the conditions for people with disabilities like, for example, children with cerebral palsy who need regular exercise, people who need regular rehab exercises. Since they’ve already stopped doing the exercise, their condition will worsen, and the same thing will happen in musculoskeletal and other disabling situations. So, the impact is really huge. I believe that there should be some mechanism, some research needs to be done or some kind of information or guidelines need to be developed on how this community can survive or can get services during the pandemic or something like that. That’s my thought.”* (ID30)
	4.2 The aftermath of COVID-19 will dictate how global organisations operate, which will influence global responses to healthcare.	*“I have no opinion as to the competencies of the WHO, but what we have not seen is a global joined up response to the COVID-19 crisis. I suspect that we should anticipate a very intensive revision of just how global organisations are expected to perform and behave in the coming months and years. So, preparing a global strategy* [one] *will need to be cognisant potentially of changing international organisational structures.”* (ID15)
**5. There are multiple inequities associated with impaired MSK health**	5.1 MSK conditions, pain and injury increase socioeconomic inequity in LMICs due to significant work and participation restrictions associated with MSK impairments and poor access to care.	*“I’ve done some work in Botswana … and I recognise that when the breadwinner is compromised through musculoskeletal disorders then that has a real ripple effect throughout the whole family and into the community. I think particularly in areas where the social determinants of health are such that citizens are poorly supported, an inequity leads to an exacerbation of these musculoskeletal disorders.”* (ID7) *“It is a big issue. For example, people with arthritis or who have bone problems, they experience huge pain, but they are still struggling to work. Even like, for example, if you don’t have some noticeable symptoms, employers don’t give you permission to go and take rest, so they keep pushing you to work even though you have those kinds of musculoskeletal problems.”* (ID30)
**6. Service delivery for MSK health is characterised by multiple complexities**	6.1 Delivery of healthcare for MSK health is complex and perhaps more complex thanother health conditions. This is owing to the wide range of health professionals who manage people with MSK health impairments and often the requirement for a continuum of care over a protracted period. This breadth makes care co-ordination challenging, navigation of the health system by patients difficult, and developing systems reform initiatives inherently more complex.	*“there is a lack of continuation of care from the inception to eventually the late stage, both in musculoskeletal and pain …”* (ID4) *“… there is the fact that for all these other chronic conditions that you have said, a specific physician is treating them. In musculoskeletal conditions, apart from the fact it can be a physiatrist or as it happens in some countries directly a physiotherapist that can treat them, it can be a rheumatologist, it can be an orthopaedic surgeon. It’s also sometimes really difficult for the patient to understand who should take care of them.”* (ID29)

*HIV/AIDS* human immunodeficiency virus/ acquired immunodeficiency syndrome, *LMICs* low and middle-income countries, *MSK* musculoskeletal, *NCDs* non-communicable diseases, *WHO* World Health Organization

#### MSK health is afforded a relatively lower priority status compared with other health conditions and is poorly legitimised

The strongest contextual challenge identified by KIs, across all economic bands, was that MSK conditions are, and historically have been, afforded a relatively lower health priority status across all levels of society and governments. MSK health is rarely found to be granted a legitimate place in policies, directives, budgetary allocations or priority statements. Three specific issues were deemed relevant to the lower priority status:

i. MSK health conditions are considered a lower priority compared to other conditions more closely associated with mortality and urgency.
ii. MSK pain and specific MSK conditions are often poorly understood, recognised, measured, treated and legitimised in policy, practice and in community attitudes.
iii. Underinvestment in service delivery, workforce and research.

i) *MSK health conditions are considered a lower priority to other conditions more closely associated with mortality and urgency.*

The strongest issue to emerge was that governments have traditionally, and continue to, prioritise health conditions more closely associated with mortality and urgency (such as cancer and CMNN diseases) over MSK health.

*“Musculoskeletal diseases have been traditionally an area of low priority in medicine, have been low status and the reasons for that, again, is that you’re not saving lives, it’s no blood, it’s no drama”* (ID2)

“[MSK] *has not been and is not yet a priority at the very high governmental level. So, for me, the simple answer is there is a lot more to do to see results in the world … in general, public health has always ignored musculoskeletal health in the last ten years in the rich countries, and in the middle- and low-income countries you have very little.*” (ID4)

KIs expressed frustration that lower priority status was primarily attributed to the relative low mortality rate associated with MSK conditions and that the disability burden alone attributed to MSK conditiond, pain and injury was not sufficiently compelling for governments to act, despite the broad-reaching health, social and economic impacts. As a result, MSK health is often ‘forgotten’.

“*I think musculoskeletal would need to compete with so many other priorities that low- and middle-income countries are faced with, but I think the important difference here is you can show a very high number on mortality on so many* [other] *NCDs and even communicable diseases. The mortality number is missing* [for MSK], *although there is a tremendous burden of disability and other things that we can talk about. But I think the sheer fact that there is no hard number on mortality that you can count, it just slips very low on the priority side.”* (ID19)

KIs from high-income countries (HICs) were pragmatic about MSK health being a lower priority in LMICs due to the burden of CMNN disease priorities.

*“Priorities in low-income countries are in mother and infant mortality, high birth rates, lack of birth control, infectious disease, malaria. They have huge, huge burdens to overcome, so I can understand why musculoskeletal disorders may not be their top priority.”* (ID22)

Emphasis was placed on the need to more clearly articulate the burden of disease associated with MSK health and increase awareness of MSK conditions, particularly at government levels.

*“I’m not sure at the moment, even now, whether we’ve done a good enough job in emphasising the economic burden of musculoskeletal disorders to governments and demonstrated well enough the potential benefits of investment in terms of disability prevention. So, I think that’s also an important area … But I think the cost is not just simply in terms of losses from a psychological and functional perspective to that individual. I think it’s important to recognise that musculoskeletal disorders present a huge burden to families, to communities, and on a societal perspective, as well”.* (ID7)

ii) *MSK pain and specific MSK conditions are often poorly understood, recognised, measured, treated and legitimised in policy, practice and in community attitudes.*

KIs from HICs highlighted that chronic primary MSK pain and chronic secondary MSK pain (e.g. specific MSK conditions and injuries), classifications defined by Treede et al. for ICD-11 [48, 49], are poorly understood, recognised and legitimised by the community, clinicians and within health systems, which could also be a contributing factor to the relatively low status of MSK health conditions.

*“Musculoskeletal pain, musculoskeletal dysfunction have not been recognised as being important or even particularly recognised in terms of being costly. We see it in pain studies about the costs of these things and sometimes there is some literature about it, but it hasn’t reached political awareness that this is a major cost to our healthcare system and it hasn’t really reached the gatekeepers of the medical care system, which in North America are generally most of them MDs* [medical practitioners], *but they’re trained in the allopathic system.”* (ID18)

In particular, the invisible nature of chronic MSK pain and lack of a ‘specific’ diagnosis can leave patients feeling helpless and frustrated that their condition is not being recognised.

*“One of the other problems is that pain doesn’t have a home. Everything is very specific to a specific diagnosis, osteoarthritis or rheumatoid* [arthritis]*, everything is very specific, there’s nothing for just “pain”. So many people are lost, especially if they don’t have a specific diagnosis, so I think too talking about musculoskeletal pain more in the general conversation or in those societal narratives is a really important thing, so that people can know that it’s real and they can be affirmed and feel acknowledged and validated.”* (ID8)

KIs identified that while chronic pain has historically been poorly measured, the implementation of the new ICD-11 classification system for chronic pain presented an opportunity to address knowledge and pain classification gaps for chronic primary pain, in particular, allowing greater characterisation and measurement of pain conditions among people who access and monitor health services.

*“Well, the ICD-11 codes, they do offer, I think, a major step up in our ability to do it* [measurement]*, particularly for healthcare contacts. I think that’s really classification. When people have come into contact with the healthcare system and that data is captured, I think that is what I see as the primary benefit. So, I think the nomenclature around the pain codes is good because you’ve got primary pain and you’ve got the ability, therefore, to better characterise pain where you can’t attribute it to something else, but you can also have a secondary pain code when there is a primary condition that drives it.”* (ID21)

iii) *Underinvestment in service delivery, workforce and research.*

Given the lower priority status and poor understanding and recognition of MSK health, there has been less investment by governments and donors in service delivery, workforce and research.

*“These disorders are the most widespread, expensive and disabling health care problems yet global priorities barely mention them, and they are very low on the priority list of most governments and charities. The IHME ‘Financing Global Health document - Developmental Assistance for Health’ described the financial and in-kind contributions provided by global health channels to improve health in developing countries. In 2015, $36.4 billion in Developmental Assistance for Health was disbursed. Only $475 million was devoted to non-infectious disease and musculoskeletal disorders was not even mentioned.*” (ID10)

#### Improving MSK health is more than just healthcare

KIs across economic bands identified a lack of recognition that MSK health is relevant beyond healthcare and extends to other important areas such as industry and workplaces, environment, social support, transport and infrastructure. Several KIs emphasised the need for a multi-sectoral approach by national governments to integrate MSK health into public policy to drive population improvement in MSK prevention and management, ideally through inter-ministerial co-operation.

*“But it’s right from the top of leadership at government when you’re going through the healthcare delivery system and wider in employment, so all the various government departments, you see that musculoskeletal conditions are recognised as one of the potential risk factors that they need to take on board, because it has an impact on not only health outcomes but also the economy and a whole range of other stuff.”* (ID17)

In particular, the built environment (residential and commercial buildings, road and transport systems and open spaces) was cited as not being conducive to optimising MSK health and supporting people with mobility limitations to function and participate and for children to play.

*“People who do not have secure housing, who do not have access to nutritious food, who do not have safe places to recreate and move, it’s not like they’re just making choices to not change their lifestyle; their environment is prohibitive of them being able to change their lifestyle. So, there are things that can be done to change that too, like created environments, built environments can go a long way towards including musculoskeletal health that aren’t ever going to be done in the clinic, they have to be done in the community.”* (ID8)

### Global guidance is needed for country-level health system strengthening

KIs advocated a clear need and value for a global strategy to guide improved prevention and management of MSK health and articulate longer-term strategic planning and directions for country-level system strengthening responses.

*“But I think that raising awareness in whatever form is critical if we are to gain any sort of success when it comes to musculoskeletal disorders. We need to raise that awareness and without a global strategy I think the management of musculoskeletal disorders will continue to be suboptimal, it’ll continue to be relegated. So, I think just merely stating that there’s a problem is not the answer. I think we all know there’s a problem and just mitigating that is not the answer. So, I think any action that is taken needs to be significant and it needs to be sustained...It’s very easy to just have a campaign and then put it away and forget about it and then the problem just carries on.”* (ID7)

However, a critical consideration with the development of a global MSK strategy was that global guidance had to be adaptable and flexible to individual countries’ priorities and context (as reflected in the guiding principles of the logic model; category 3). It was strongly identified by KIs both from HICs and LMICs that a strategy developed for one context, e.g. a single country or economic band, would not be transferable to all countries. For this reason, a global strategy needs flexibility and adaptability to unique country-level contexts.

“*… so a specific musculoskeletal policy which will cater now for all the needs, like the issue of mobility, the issue of access. Yes and that I think can be really actually promoted at a global level and then it’s for the individual governments worldwide to decide what level they’re able to adopt, according to their resources and political will.”* (ID3)

*“You can’t take a strategy from one country and just implement it in another country.” (ID4)*

In developing a global MSK strategy, a few participants warned that the political window for drawing attention to MSK health and pain care is narrow. While external framing of the argument is important, it needs to extend beyond just the disability burden and more explicitly convey costs and return on investment from addressing disability and deaths from injury and trauma, particularly to governments.

*“What is the political window that musculoskeletal conditions can take advantage of as an entry point in the discussion? I think that is really one of the critical points and one of the critical difficulties in the discussion of musculoskeletal conditions because I don’t think that it is possible anymore. Of course, one can advocate still for the SDGs and SDG-3 and Universal Health Coverage and so on, but something else needs to come into the narrative, otherwise this narrative has been used already too broadly and too often. Potentially, it is exclusively the narrative of the cost that is unique to the window of opportunity that has demonstrated that the cost is extremely high. That is something that we don’t want, so we need to do something so that the costs are reduced, especially then when other countries come into the transition of really having more and more people with musculoskeletal conditions.”* (ID20)

Global leadership from the WHO in positioning and prioritising MSK health was also considered extremely important to catalyse and sustain a global response to the burden of disease, particularly in LMICs and the strategic directions of global clinical organisations. A WHO global strategy would assist Member States to initiate appropriate policy, financing and health service reform initiatives and for clinical organisations to prioritise their efforts in global reform initiatives.

*“For example, if one strategy is developed by some other international organisation, for example, International CSO* [Civil Service Organisation] *the government might consider it, but if it is through the WHO they unconditionally accept it and they work wonders in achieving that strategy, implementing that strategy. I can even mention an example. In Ethiopia, rehabilitation was not part of the health system, it was under the Ministry of Social and Labour Affairs. But now, because of the push from the WHO in making rehabilitation part of the health system, they are trying to do some changes and as of last year rehabilitation became part of the health system...”* (ID30)

#### COVID-19 will have an impact on MSK health globally and opportunities for health system strengthening

KIs, predominantly from HICs, anticipated that the COVID-19 pandemic would further exacerbate the relative low priority afforded to MSK health and likely widen care disparity gaps for MSK conditions and MSK pain care in vulnerable groups. It was also anticipated that the disability burden from MSK conditions due to COVID-19 would increase, for example, due to a decrease in physical activity and social isolation.

*“I think the COVID thing for us in North America and Europe, at least, has put musculoskeletal care on the backburner...- I’ll use Canada as an example. We were projecting a $20 billion federal government deficit this year* [2020]. *It’s now $300 billion as a deficit. So, there’s a factor of 15 times greater deficit than anticipated, which means that funding for musculoskeletal health and musculoskeletal health research is going to be very much diminished and I think that’s going to be true all across the world. I think there’s going to be a profound lack of government support for musculoskeletal illness. They’re going to focus first and foremost on COVID, obviously, but then they’re going to continue to focus their attention and their remaining financial resources on what they consider life-threatening illnesses.”* (ID26)

The COVID-19 pandemic also has highlighted the need to re-think how global and international organisations perform to support health reform. For instance, a KI indicated that in developing a global MSK strategy, awareness of potential future shifts in international organisational structures is needed.

*“… what we have not seen is a global joined-up response to the COVID-19 crisis. I suspect that we should anticipate a very intensive revision of just how global organisations are expected to perform and behave in the coming months and years. So, preparing a global strategy,* [one] *will need to be cognisant potentially of changing international organisational structures.”* (ID15)

### Category 2: Goal

The goal reflected the intended purpose of the project and incorporated features of the empirically-derived Guiding Principles (category 3).

### Category 3: Guiding principles underpinning a strategic resoponse

Five guiding principles were derived, largely reflecting the challenges in addressing MSK health at a global level:

1. **Adaptability**: global guidance and recommendations must be adaptable to local cultural, political and economic contexts.
2. **Inclusiveness through co-design**: global guidance and recommendations must be co-designed through consultation across economies, intentionally including people with lived experience of MSK health impairment conditions and vulnerbale populations.
3. Reduce disability to **improve function**, quality of life and overall health.
4. Adopt a **life-course approac**h to MSK prevention and management.
5. **Equity and value-based care**: prioritise equitable and early access to the right MSK health care and de-adopt low-value care.

These guiding principles are summarised with supporting quotes in Table 3.

**Table 3:** Guiding principles for a global MSK strategy

Principle	Description	Illustrative quote(s)
***Adaptability*: global guidance and recommendations must be adaptable to local cultural, political and economic contexts.**	A strategy for implementation in every country is neither possible nor appropriate. Goals or actions within a global MSK strategy need to be broad and adaptable to the local context to take account of beliefs, cultural sensitivities and economic contexts that are unique to each country.	*“… but different countries are going to have unique issues that are going to be really hard to address with a global strategy, but the global strategy I think can encompass enough that there’s flexibility for each of those individual regions or countries, or regions within countries.”* (ID7) “*You can’t have a “global” education program. It has to be context-specific. In the USA we might be busy saying to people, “Look, from an education point of view, this is what we need to do to allow you to be able to go to the tennis club” but from a low income point of view, “This is what we need to be able to do so that you can walk to go and get your water” and in the middle income to walk and go and get your shopping, get your necessities for the day or to be able to work and earn money for your family. So, it’s those kinds of context-specific things that we need to think about.”* (ID25)
***Inclusiveness through co-design*: global guidance and recommendations must be co-designed through consultation across economies, intentionally including people with lived experience of MSK health impairment and vulnerable populations.**	Components of a strategy should be underpinned by robust consultation and co-design approaches to ensure representation and meaningful engagement and inclusiveness across economies (in particular, not dominated by perspectives of high-income settings) and intentionally include people with lived experience and vulnerbale or marginalised groups. This approach should also and enable local implementation strategies to be devised to suit the local context and population needs.	*“I think that really being intentional about who creates that, because things tend to be very Western dominated, very wealthy country, kind of, dominated, very white dominated, all of those things, and patients and community members can be excluded from the creation of those things. I think it misses a lot when those groups aren’t involved in the creation of it because they’re the ones that are then supposed to live it out, but they didn’t have any say in the process.”* (ID8) *“... so there needs to be an enormous amount of consultation to ensure that whatever is developed is going to be useful in local settings.”* (ID12)
**Reduce disability to improve function, quality of life and overall health.**	A key focus should be on enabling participation and function of people and communities across the lifecourse by preventing and reducing the disability burden associated with MSK health impairment(s), thereby improving quality of life and overall health. For older people in particular, this means improving locomotor capacity (mobility) as a component of intrinsic capacity to improve functional ability.	*“I think our ultimate goal should be reduced burden of musculoskeletal disorders in the global community in terms of incidence and prevalence, disability reduction, achievement of remission and relative freedom from adverse effects of drugs, improved quality of life and, ultimately, abolition of the disease.”* (ID5) *“People want to stay at home, they don’t want to be in residential care facilities, so what how can we create an environment that an older person’s mobility is the key goal? Because if mobility was a key goal then pain would be addressed, as would impairment.”* (ID23)
**Adopt a lifecourse approach to prevention and management.**	Improve MSK health outcomes across the lifecourse, with an emphasis on prevention. This is critical in acknowledgement of the impact of MSK health impairment on children’s development and function and the importance of MSK health in intrinsic capacity in older people.	*“I think the goals for prevention would be to maximise musculoskeletal health and the goals for management would be to minimise the burden and maximise function and participation as much as is reasonably possible. I also think it really does need to have a lifecourse approach, because we know that a lot of these problems track from the second decade of life into adult life and that the exposures are often cumulative over a long period of time, as I think is the burden over time.”* (ID21) *“… educate people that actually play* [for children] *is important for your long-term health. People kind of know that but they’re not thinking about musculoskeletal, they’re thinking about heart disease and diabetes.”* (ID6)
***Equity and value-based care*: prioritise equitable and early access to the right MSK health care and de-adopt low-value care.**	Prioritise equitable and early access to the right MSK and pain care (i.e. care that is safe, effective, affordable and acceptable to patients) and de-adopt low-value care options. For LMICs and some high-income countries, this will require improving access to vulnerable groups to reduce equity gaps. Here, low-value care refers to care that is not supported by evidence, is not cost effective and has the potential for harm, or the risk of harm exceeds probable benefit [50].	*“I think the priority has to be equitable access to care and is critical in management strategies and, again, I understand that in low- and middle-income countries health equity is a massive, massive issue. With so many parts of the world that don’t have any access to health services at all, musculoskeletal will come even further down the list probably than it would have been before, but we need to prioritise equitable access to care.”* (ID7) *“So, I think for the global community and particularly at a high level, there is a need to better reinforce evidence-based treatments that are culturally adapted....”* (ID4)

*LMICs* low and middle-income countries, *MSK* musculoskeletal

### Category 4: Accelerators

Accelerators represent specific supports necessary to catalyse and sustain the development and implementation of a global strategy for MSK health. This terminology has been described previously in the context of creating value-based health systems [39]. Seven accelerators were identified by KIs (Table 4 ). The three major accelerators to emerge, are discussed below.

**Table 4:** Accelerators for the development and implementation of a global MSK strategy

Accelerator	Description	Illustrative quotes
**Leveraging multi-sectoral partnerships and cooperation to facilitate sustainable and scalable change**	A multi-sectoral approach that supports engagement and education of the community (people, patients, organisations, governments) and the establishment of partnerships between government and non-government agencies (including existing regional societies) to address prevention and management of MSK health is needed. Additionally, consideration of cultural differences in how health is conceptualised is necessary. To achieve the necessary scale of change, this strategic approach must extend beyond the healthcare sector and intentionally and explicitly involve multiple other sectors of the community and across government ministries.	*“I think there has been general momentum in the last 5 to 10 years for existing rheumatology or musculoskeletal associations to communicate much more clearly with each other to think about working together, to think about a much more collective approach. Certainly, that is a direction that EULAR has really taken very seriously and we now have improving relationships. We’ve, for some number of years, had strong relations with American colleagues, but I have to say we’ve worked very hard to build strong relationships with the Asia-Pacific League, with PANLAR and, especially in the last short number of years, with AFLAR to really seek that. Obviously, there are low- and middle-income countries in all of the regions, but we’ve already started to look at building consensus.”* (ID15) *“I think it needs to be targeted, anything we do needs to be targeted at all levels. I think targeting at governmental levels is one, but I think civil society, public health agencies, employers, but also community awareness and community help. So, I think any campaign would need to be targeted at all levels in order to be effective.”* (ID7)
**Intentional alignment with existing global or international strategies and initiatives**	An MSK global strategy needs to align with, and link into existing global strategies and initiatives that are relevant to a lifecourse approach. There is a need to focus on health and participation that extends beyond MSK health (e.g. healthy ageing, NCD prevention and control, obesity management, rehabilitation, physical activity, work), including SDG-3, and be formulated in a way that can align with existing national strategies.	*"I guess you’ve got to go back to make sure that you’re connecting with the Clinical Consortium on Health Outcomes at the WHO and you’ve got to be knowing that the Integrated Care for Older People (ICOPE) is evidence-based. So, you’ve got specific plans and programs already happening." (ID23)* *“… I also think that there needs to be a willingness to link our strategy with existing strategies outside of musculoskeletal health as well, because there are so many crossovers. Obesity is probably one that comes to top of mind and obesity is an issue with cardiovascular health and cancer and many others as well. So, we need to make our strategy, it needs to have the ability to overlap and co-exist with other strategies …”* (ID12)
**Identify essential, evidence-based standards or actions to enable lower-resourced settings to initiate action on MSK health**	A strategy should identify the minimum (essential), evidence-based actions and standards for effective prevention and management of MSK health conditions, pain care and injury care. Depending on resourcing, priorities and context of each individual country, there may be options for advanced (desirable) standards or care considerations. This framework would provide guidance on ‘what’ essential care to provide, allowing countries to decide ‘how’ best to deliver the care in their unique contexts.	*“The concept of this being a universal thing is difficult. Things that are relevant to Australia, Canada, United States and Great Britain are largely irrelevant, I think, in some low- and middle-income countries, other than the philosophy of care. So, I think the emphasis should be more on the philosophy of care in terms of what’s important and what isn’t important, as opposed to the mechanics of care, if I can use that term.”* (ID26) *“One thing I think from a simple rehabilitation perspective is to create sort of packages of rehabilitation interventions that should be done for the management of musculoskeletal conditions. Like a minimal level of assistance that should be provided in Universal Health Coverage for musculoskeletal conditions and a sort of recipe of the minimal amount.”* (ID29)
**Increase public and government awareness of MSK health, the impacts of MSK-related disability and that effective management is possible**	In order for multi-sectoral reform initiatives to be effective and sustainable, there is a need to increase and improve the awareness among society, policymakers and global organisations in relation to: ○ the importance of MSK health; ○ the socioeconomic impacts of MSK-related disability on people and broader communities; ○ the management of MSK conditions and that effective care is possible, supported by data and actions/solutions.	*“I think one thing is to follow the route that all major global health problems have taken, that is to raise awareness, show data, show the impact of the problem and, at the same time, propose a solution. So, I think it’s a package that we need to present to the international community, to the decision-makers, politicians and global health agencies”* (ID19) *“From a personal perspective I think we need to move musculoskeletal health away from a somewhat ethereal area of rheumatism, perceived to be a disease of the older population, to something that is actually amenable to modern molecular, digital, AI-driven interventions that can really change lives and that can really inform policy, that can keep people working, that can keep people with their families for longer.”* (ID15)
**Co-design objectives and performance indicators relevant to desired outcomes and implementation**	A global strategy needs to be accompanied by an action plan that articulates specific objectives and performance indicators that are co-designed by the global community through thorough consultation. Objectives and indicators must be acceptable and feasible to guide implementation activity, resourcing and monitoring, including evaluation of implementation. This is relevant at the global level and to support development at the country level.	“*That would be the starting point to actually lay out what the expectations are within the strategy and having the supporting action plan to actually then understand what needs to happen. And then the measurement as well of that, a system, so it’s not just an action plan that sits on the shelf and never comes to fruition, but actually having systems in place where that’s then measured in a timely way.”* (ID17) *“… but I thought that what you hadn’t sorted out was what it was you were trying to reduce. So, to me, the aim of a musculoskeletal strategy is to reduce fractures, to reduce hip replacements, to improve WOMACs, to reduce VAS scores for back pain.”* (ID9)
**Language translation is essential for improving access and uptake**	To support dissemination and uptake of a global strategy for MSK health, investment in translation beyond the six official UN languages is important, to ensure that all countries have access to global guidance.	*“So, I think that language barrier is an important issue, especially when I think about global strategies. When translations are provided they are in French and Spanish. Other languages, like Brazilian Portuguese, Portuguese from Portugal, German and Italian, are not covered … So when we plan or develop a strategy with global impact we need to overcome this language barrier, providing the most translations that we can, like Japanese and Arabic. So, we need to overcome and also provide strategies that are inclusive and not only in English and assuming that everybody can read in English.”* (ID28)
**Provide guidance on MSK health care in the context of pandemics (e.g. COVID-19)**	A contemporary global strategy for MSK health needs to consider the impact of COVID-19 (and potentially future pandemics) on access to MSK healthcare services and the likely impact on COVID-19 on MSK health. In this context, providing guidance on how countries can manage MSK health in the context of a pandemic is important; e.g. guidance on self-management, guidance on access to rehabilitation services.	*“...then because of COVID there are so many restrictions to, for example, physical exercise and things like that so we encourage people to do the online exercise and online self-management. So, it’s very important to have that aspect and also self-care and self-management, not always prescribed by physiotherapists or doctors, it’s more like how they can adopt healthy behaviour to promote mobility and decrease the pain and cope with pain.”* (ID24). *So, because of the COVID situation, actually government is trying to narrow down what is an essential service for babies, essential for adults and essential for the older adults.” (ID24)*

*AFLAR* African League of Associations for Rheumatology, *EULAR* European Alliance of Associations for Rheumatology, *LMICs* low- and middle-income countries, *ICOPE* Integrated Care for Older People, *MSK* musculoskeletal, *NCDs* noncommunicable diseases, *PANLAR* Pan-American League of Associations for Rheumatology, *SDG* Sustainable Development Goal, *VAS* visual analogue scale, *WHO* World Health Organization, *WOMAC* Western Ontario and McMaster Universities Osteoarthritis Index

#### Leveraging multi-sectoral partnerships and cooperation to facilitate sustainable and scalable change

The strongest accelerator identified was the need for a multi-sectoral approach that supports engagement and education of the community (people, patients, organisations, governments) and the establishment of partnerships between government and non-government agencies (including existing regional societies) to address prevention and management of MSK health. This was predominantly discussed by KIs from HICs.

*“I think you’ve got to look at the structure of how you’re going to do this. We need a multisectoral/multidiscipline approach and that needs to be central to any action plan … so it’s the relational building that’s required, the word “intersectionality”, what are the common values? And it does come down to values.”* (ID23)

Also advocated, was the need to consider cultural differences in how health is conceptualised across Member States. To achieve the necessary scale of change, any strategic approach must extend beyond the healthcare sector and intentionally and explicitly involve multiple other sectors of the community and across government ministries. KIs identified the need to work with regional societies and their existing advocacy and program initiatives to build a coalition of support.

*“Policymakers, politicians, healthcare planners, but also people involved in higher education. Think about the workforce. There are also people who are involved in town planning. I mean, that’s all policy too, but helping to include people in all aspects of their life, whether they’re disabled or not, impacting people’s independence. So yeah, I guess it’s policy, politicians, healthcare planners, higher education, school planners.”* (ID6)

*“So, a global strategy would need to be judiciously created and would need to work very, very closely with the existing regional societies who are long-established and have built up their own infrastructure, networks, connectivity and, in some cases, very strong advocacy programs, so anything that emerged would need to be very complementary to what was already there.”* (ID15)

#### Intentional alignment with existing global or international strategies and initiatives

Participants were unequivocal in their view that an MSK global strategy should link with existing international and global MSK initiatives and initiatives that extend beyond MSK health, whether they be developed by regional professional associations, international non-government associations or global organisations such as the WHO. For example, healthy ageing, NCD prevention and control, rehabilitation, and physical activity to complement SDG-3.

*“… I also think that there needs to be a willingness to link our strategy with existing strategies outside of musculoskeletal health as well because there are so many crossovers. Obesity is probably one that comes to top of mind and obesity is an issue with cardiovascular health and cancer and many others* [NCDs] *as well. So, we need to make our strategy, it needs to have the ability to overlap and co-exist with other strategies and that would not be hard to do but, once again, that’s going to come with a level of engagement across not only musculoskeletal health, but other health areas as well.”* (ID12)

KIs also highlighted that there was a need to link into existing systems and structures to monitor MSK conditions globally.

*“I think it’s really important that it’s informed by the work the WHO does more broadly, because what I think we need not to do is to come up with something that doesn’t sensibly integrate with other initiatives that they’ve got underway. Because we know that there will be a set of things, say, in terms of prevention that are cross-cutting and where the surveillance systems are already in place and really, we just need to make sure that we monitor musculoskeletal conditions as part of that.”* (ID26)

#### Identify essential, evidence-based standards or actions to enable lower-resourced settings to initiate action on MSK health

Recognising the vast differences across countries in terms of resources, priorities and context, it was strongly emphasised by KIs that a global MSK strategy should identify the minimum (essential), evidence-based actions and standards for effective prevention and management that could be adopted by all countries.

*“The other part means you can address, and there’ll be some flexibility here rather than being prescriptive, if you can design an intervention which can be addressed either as desirable or essential. So, essential means we will expect that, regardless of the level of income, economic level of a country, we believe that, say, physiotherapy should be available at the secondary care level. If we can get it at primary level it’s desirable, but at secondary level it has to be essential. So, I think the other way is to differentiate interventions as essential and desirable across the hierarchy of health systems.”* (ID19)

After establishing essential standards or care considerations, KIs, particularly from LMICs, indicated that options should also be provided for advanced (desirable) standards or care considerations that individual countries could choose to adopt and/or adapt.

*“One can have one goal, but I was thinking that maybe we need to have a two-tier goal where one would be this would be the basic minimum we’ll be expecting and then we would have the graduated one, which is the upper level, and then it is for every country to assess itself where it fits in..”* (ID3)

## Discussion

Despite consistent data highlighting the profound burden of disease and socioeconomic impacts attributed to MSK conditions, pain and injury/trauma, why are health systems strengthening efforts lacking and how should this be addressed at a global level? Purposively sampled KIs, irrespective of their country’s economic development, strongly endorsed the value proposition of a global response to improving country-level prevention and management of MSK health through thoughtful co-design of a global strategy, ideally championed by the WHO and intentionally integrated with current and emerging initiatives in NCDs, ageing, disability, rehabilitation and injury and trauma care. KIs consistently identified that MSK health is afforded a lower priority status relative to other NCDs, that improvements require action beyond direct healthcare and that the COVID-19 pandemic will likely slow progress in health systems strengthening despite the likelihood of COVID-19 impacts on MSK health. They opined that global guidance on MSK health would positively influence socioeconomic inequity, particularly in LMICs by acting on the global burden of disability, and provide country-level guidance in systems strengthening, yet cautioned that implementation priorities will necessarily vary by country: “*You can’t take a strategy from one country and just implement it in another country*”.

KIs strongly identified the need for MSK health to be explicitly integrated with other NCDs into policy, financing, workforce and service design initiatives. This was seen as particularly important for LMICs, where establishing new or stand-alone programs was viewed as inappropriate given the multiple, competing health demands and low resourcing in those settings. Rather, integration was preferred in these settings, consistent with the principle of development effectiveness [30]. However, tension exists with current policy foci for NCDs focused on cancer, cardiovascular disease, diabetes and respiratory conditions. While these condition foci are relevant and important for reducing premature mortality, particularly in the context of targets for SDG-3, the relative lack of policy attention to MSK health starkly contrasts contemporary burden of disease profiles and costs to health systems [4, 22, 29, 33]. KIs identified this challenge of raising the policy priority of MSK health, given the lower mortality burden compared with other NCDs and advocated the need to articulate data-driven arguments to governments concerning the health and economic burden of disability. However, evolving health priorities and performance indicators from a mortality reduction target to a broader measure that considers functional ability, and therefore MSK health as a central tenant, will remain challenging. The SDG-3 target, current foci of the WHO Global action plan for the prevention and control of NCDs 2013–2020 and the Political Declaration of the Third High-level Meeting of the General Assembly on the Prevention and Control of NCDs all focus on premature mortality [6, 51]. Nonetheless, there are promising policy signals to support MSK health prevention and management, for example from OECD Member States and recommendations for GBD 2019 [4, 29]. KIs identified the need for a clearer understanding of MSK health and specific MSK conditions. In particular, a more contemporary understanding of chronic primary MSK pain and better surveillance of pain conditions through implementation of the ICD-11 system [52].

KIs identified that MSK health, like all health, is dependent on factors beyond physical and mental healthcare such as industry, the built environment, transport and other social determinants, necessitating a multi-sectoral approach to reform. Indeed, recent evidence points to the relevance of social determinants of health for MSK health outcomes [53, 54]. The importance of engaging the community in co-design of residential and commercial buildings and open spaces to facilitate access, function, physical activity and play was strongly advocated. This perspective mirrors that of the UN General Assembly [55] and themes identified previously in policy for integrated prevention and control of NCDs [29]. MSK health is dependent not only strengthening within these sectors (intra-sectoral), for example minimising road traffic trauma through recommendations in the World Report on Road Traffic Injury Prevention [56], but also approaches that integrate between sectors (inter-sectoral), for example, the WHO age-friendly cities and communities network [57].

All KIs strongly supported the need for, and potential value of, a global strategy to guide improvements in prevention and management of MSK health, underpinned by five guiding principles. Critically, KIs identified the need for any strategy to be co-designed with multi-sectoral stakeholders (including people with lived experience) and to be relevant across the lifecourse. KIs advocated for a strategy that targeted function through the adoption of high-value care and de-adoption of low-value care with reform priorities that are adaptable to local contexts in recognition of variability in economic development and health priorities across countries. These attributes align with the principles of developing system-level models of care and transformations needed to deliver high-value care in health systems [39, 58, 59].

Critically, KIs observed any strategy must intentionally align and link with existing regional and global strategies, especially at the level of the WHO, perceived by KIs as the essential champion for any global health strategy. Here, aligning with existing and emerging WHO programs and strategies in NCDs, ageing, child and youth, disability and rehabilitation, and injury and trauma was deemed critical, especially in LMICs. Identification of essential packages of care for MSK health that have applicability irrespective of economic development was also identified as a key accelerator. Such an approach would provide countries with guidance on minimum essential prevention and management practices and also inform a suite of other contextually-appropriate options. Indeed, this approach aligns with the current WHO Package of Rehabilitation Interventions initiative and would better facilitate integration of MSK health services into UHC packages [60].

The rapid change in the global health landscape from COVID-19 presents an immediate challenge and imperative for co-development of a global strategy for MSK health. While the impact of COVID-19 will likely mean less resourcing and prioritisation for NCDs in the short-term, the post-pandemic era will likely be characterised by an increasing need to address NCDs exacerbated by COVID-related illness, including MSK conditions, the sequelae of reduced access to non-acute healthcare and social impacts of the pandemic, and ‘long COVID’ presentations [61].

Strengths of this research include a large and diverse sample of highly experienced KIs from all UN regions, sampling across all levels of economic development and across multiple sectors. Further, we purposively sampled across clinical disciplines and from peak global and regional organisations. Valuable perspectives from consumer advocates and people with lived experience highlight the critical importance of person-centred viewpoints [58]. The logic model provides a data-driven and contemporary framework upon which a global strategy can be formulated. This framework fills an important gap in health systems strengthening, where relative to conditions more closely aligned with mortality [62–65], mental health [66] or lifecourse [67, 68], data-driven global guidance for systems reform in MSK health is lacking. The intentional co-design approach for the logic model and later planned stages of this program of research facilitates stakeholder buy-in from inception through to implementation. Further, the logic model aligns well with a recent framework for creating value-based health systems and the principles articulated by the UN General Assembly [39, 51], providing a level of construct validity. Relevant study limitations include relative over-sampling of KIs from clinical/professional organisations and from Europe with less representation from Africa, Oceania and Latin America. The sample was also under-represented by younger KIs, by representatives from national Ministries of Health, and by clinicians working specifically in child and adolescent health. While KIs represented 25 organisations and redundancy in derived themes was achieved through phased recruitment and analysis, we can neither assume that the perspectives of other relevant stakeholder organisations are reflected in the data, nor that the data reflect the broader or endorsed views of the represented organisations.

## Conclusions

There is strong multi-sectoral support for a global-level strategic response to improve the prevention and management of MSK health impairment. Global guidance that is informed by multi-sectoral consultation and co-design efforts and is adaptable to local contexts is urgently needed to arrest the burden disability attributed to MSK health impairment. The data-driven logic model derived can be used as a blueprint for global health systems strengthening response.

## Supplementary Information

**Additional file 1.**

**Additional file 2.**

**Additional file 3.**

## Data Availability

All data generated or analysed during this study are included in this published article [and its supplementary information files].

## Abbreviations

CMNN: Communicable, maternal, neonatal, and nutritional
CSO: Civil Service Organisation
DALY(s): Disability-adjusted life year(s)
GBD: Global burden of disease
G-MUSC: Global Alliance for Musculoskeletal Health
HIC: High-income country
ICD-11: International Classification of Disease (11th edition)
KI(s): Key informant(s)
LMIC(s): Low and middle-income country(ies)
MSK: Musculoskeletal
NCD(s): Non-communicable disease(s)
OECD: Organisation for Economic Co-operation and Development
SDG(s): Sustainable Development Goal(s)
UHC: Universal Health Coverage
UN: United Nations
WHO: World Health Organization
YLD(s): Year(s) lived with disability
