# Supplementary material for: The need for adaptable global guidance in health systems strengthening for musculoskeletal health: a qualitative study of international key informants
Source: Glob Health Res Policy. 2021 May 28;6:24. doi: 10.1186/s41256-021-00201-7 (PMC8277526; doi:10.1186/s41256-021-00201-7)
Supplement: Supplementary file 2 — Additional file 2. [file 41256_2021_201_MOESM2_ESM.docx]

GRIPP2 short form

| Section and topic | Item | Reported on page No |
| --- | --- | --- |
| 1: Aim | Report the aim of PPI in the study | 8 |
| 2: Methods | Provide a clear description of the methods used for PPI in the study | 9 |
| 3: Study results | Outcomes—Report the results of PPI in the study, including both positive and negative outcomes | 18-32 |
| 4: Discussion and conclusions | Outcomes—Comment on the extent to which PPI influenced the study overall. Describe positive and negative effects | 33-37 |
| 5: Reflections/critical perspective | Comment critically on the study, reflecting on the things that went well and those that did not, so others can learn from this experience | 33-37 |

PPI=patient and public involvement
