# Supplementary material for: The need for adaptable global guidance in health systems strengthening for musculoskeletal health: a qualitative study of international key informants
Source: Glob Health Res Policy. 2021 May 28;6:24. doi: 10.1186/s41256-021-00201-7 (PMC8277526; doi:10.1186/s41256-021-00201-7)
Supplement: Supplementary file 3 — Additional file 3. [file 41256_2021_201_MOESM3_ESM.docx]

**Interview questions**

1. How would you describe the current state of musculoskeletal (MSK) healthcare (both prevention and management) globally?
2. In your opinion, what needs to be done now to improve the prevention and management of MSK conditions at a global level?
3. Global Strategies or Action Plans are often used, e.g. by the WHO, to draw attention and action to important health issues. Do you see value in a Strategy or Action Plan to guide a global response to improve MSK conditions?
4. What would you want to see in a global Strategy for the Prevention and Management of MSK health?
   - What should be the goals of the Strategy?
5. The WHO Global Action Plan for Prevention and Control of NCDs (2013-2020) is framed around 6 objectives. I would like you to reflect on each objective and discuss it in the context of MSK conditions.
   1. **Objective 1 is Prioritisation and advocacy**: Raise the priority of prevention and control of NCDs in global, regional and national agendas and internationally agreed development goals, through strengthened international cooperation and advocacy.
      - What would you consider to be the specific priorities to improve prevention and management of MSK conditions?
   2. **Objective 2 is Country-level system strengthening**: For individual countries to strengthen national capacity, leadership, governance, and multi-sectoral action to accelerate responses for the prevention and control of NCDs through areas such as policy, workforce and financing.
      - What would you consider to be the specific opportunities to improve prevention and management of MSK conditions at a country-level? What would be specific opportunities/priorities in the areas of:

National leadership

Citizen engagement

Policy

Workforce capacity-building

Financing

1. **Objective 3 is Risk factors and determinants**: To reduce modifiable risk factors for NCDs and underlying social determinants through creation of health-promoting environments.
   - - What should be the priorities in reducing modifiable risk factors for MSK conditions?
2. **Objective 4 is Primary care services and universal health coverage**: To strengthen and orient health systems and services to address the prevention and control of NCDs and the underlying social determinants through people-centred primary health care and universal health coverage.

- What would you consider to be the specific priorities to improve the prevention and management of MSK conditions in relation to primary care services and universal health coverage? In particular, please consider:
  - 1. Service models (or ‘models of care’) and workforce
    2. Access to essential medicines/technologies

1. **Objective 5 is Research and innovation**: To promote and support national capacity for high-quality research and development for the prevention and control of NCDs.
   - What would you consider to be the specific priorities to improve the prevention and management of MSK conditions in relation to research and innovation?
2. **Objective 6 is Surveillance:** To monitor the trends and determinants of NCDs and evaluate progress in their prevention and control, such as population health surveillance reporting and health information systems.
   - What would you consider to be the specific priorities to improve the prevention and management of MSK conditions in relation to population health surveillance reporting and health information systems?
3. Do you think any of these elements relate differently to high income and low to middle income countries?
